# The prevalence of CMV and EBV among the patients with the colorectal cancer; a molecular approach

**DOI:** 10.1186/2051-1426-3-S2-P379

**Published:** 2015-11-04

**Authors:** Amir Arastefar, Reza Ranjbar, Mohammad Amin Behzad, Kamran Dezfulian, Hamid Sharifi Mehr, Azin Aein, Mohsen Gholami

**Affiliations:** 1 Professor Alborzi Clinical Microbiology Research Center, Shiraz University of Medical Sciences, Nemazee Hospital, Shiraz, Iran; 2 Molecular Biology Research Center, Baqiyatallah University of Medical Sciences, Tehran, Iran; 3 Microbiology Department, Azad University of Rasht, Rasht, Iran; 4 Department of animal physiology, Shahid Bahonar University of Kerman, Kerman, Shiraz, Iran; 5 Family physician at Shiraz University of Medical Sciences, Shiraz, Iran; 6 Khatmaolanbia health clinic, Shiraz, Iran

## Introduction

Nowadays, colorectal cancer is known as one of the most common types of gastrointestinal cancer with high mortality rate worldwide. Recently, the association between the development of the cancer and viral infections has been widely investigated with controversial results. The current study was conducted to detect the association of CMV and EBV prevalence with colorectal cancer in the respective patients, and compare the prevalence rates of the two conditions between patients and healthy individuals.

## Methods

A total of 80 tissue blocks (58 from colon and 22 from rectum) of the patients with colorectal cancer and 80 samples (58 from colon and 22 from rectum) from normal counterparts were obtained. The block samples were deparaffinized and the viral DNA was extracted using a commercially available kit. CMV and EBV nucleic acids were detected by nested-PCR method using specific outer and inner primer sets. The results were statistically compared between different groups by SPSS for Windows (version 16, SPSS Inc., Chicago, IL, USA).

## Results

CMV DNA was detected in 7/80 (8.7%) of patients; all in colon cancer group. Furthermore, EBV DNA was detected in 70/80 (87.5%) of patients, consisting of 50/58 (5%) with colon cancer and 20/22 (90.9%) with rectum cancer. Among the healthy individuals, the CMV and EBV nucleic acids were detected in 4/80 (5%) and 65/80 (81.25%), respectively. The statistical analysis showed no significant difference between the prevalence rates of CMV and EBV infections in cancer patients and the healthy group (p > 0.05).

## Conclusion

Taken together, our findings revealed that the prevalence rates of both CMV and EBV infections were higher in colorectal cancer group than those in the normal one, though the differences were not statistically significant among them. Given the importance of the viral infections in the incidence of colorectal cancers, to reduce the infection complications in the cancer patients, a screening program is highly suggested.

**Figure 1:**
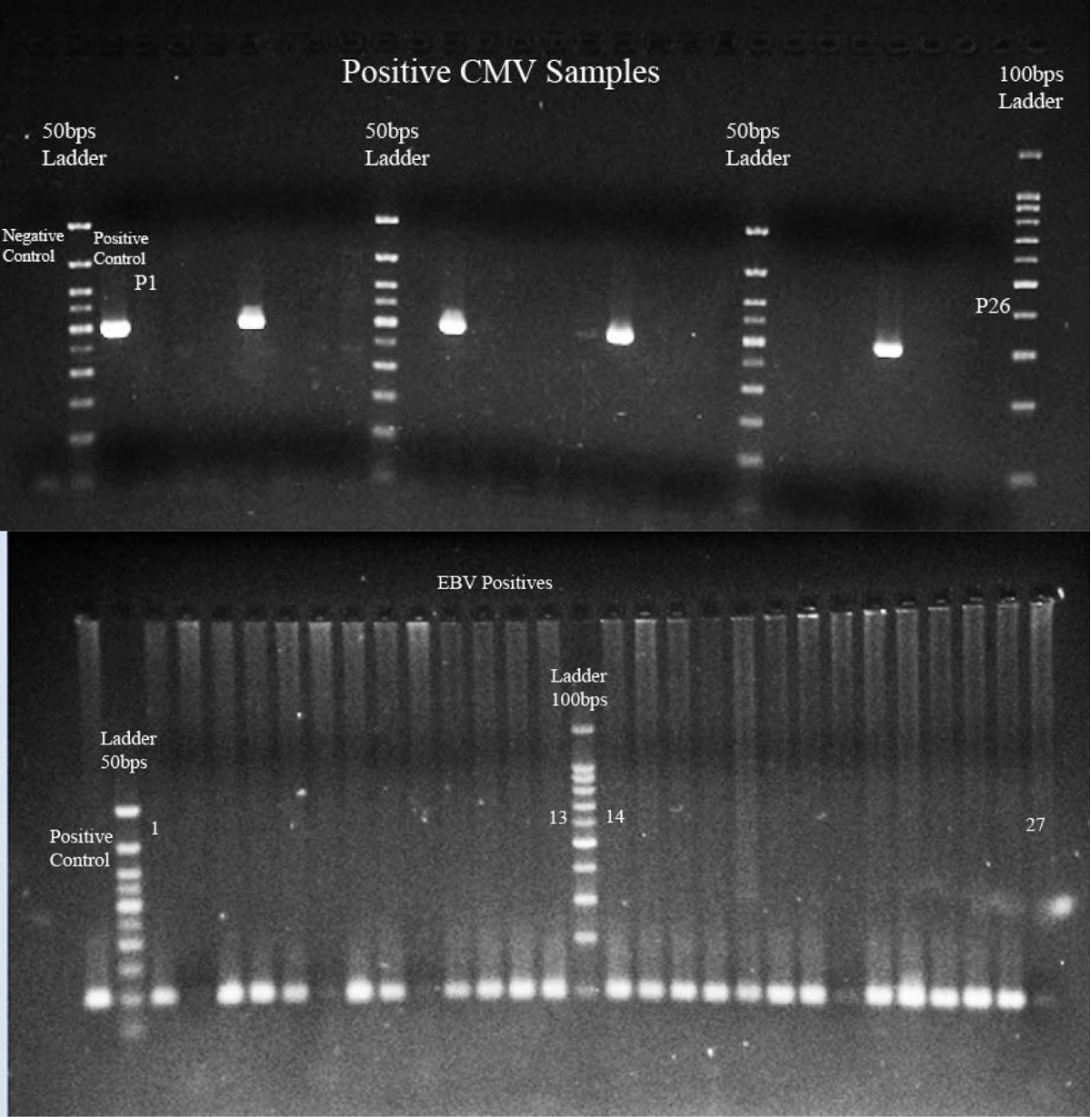


**Table 1:** Analysis of EBV infection prevalence among different studied groups.

	Variable	Frequency	p-value
**Health condition**	Healthy	65/80 (81.25%)	
	Cancer patients	70/80 (87.5%)	P=0.384

**Sex**	Male	87/100 (87%)	
	Female	48/60 (80%)	P=0.265

**Location**	Urban	80/93 (86%)	
	Rural	55/67 (82.1%)	P-0.516

**Table 2:** Analysis of CMV infection prevalence among different studied groups.

	Variable	Frequency	p-value
**Health condition**	Healthy	4/80 (5%)	
	Cancer patients	7/80 (8.7%)	P=0.534

**Sex**	Male	4/100 (4%)	
	Female	7/60 (11.6%)	P=0.103

**Location**	Urban	5/93 (5.4%)	
	Rural	6/67 (8.9%)	P=0.528

